# Case report: A rapid response to immunotherapy in a thoracic SMARCA4-deficient undifferentiated tumor with respiratory failure

**DOI:** 10.3389/fonc.2022.1020875

**Published:** 2022-11-01

**Authors:** Liyong Shi, Lianshun Lin, Yin Ding, Yiming Zeng, Xiaoyang Chen

**Affiliations:** Department of Respiratory and Critical Care Medicine, The Second Affiliated Hospital of Fujian Medical University, Quanzhou, China

**Keywords:** SMARCA4-UT, immunotherapy, tislelizumab, PD-L1 expression, immune checkpoint inhibitor

## Abstract

Thoracic SMARCA4-deficient undifferentiated tumor (SMARCA4-UT) is an extremely rare and poor-prognosis malignancy, which has recently been noted as a subtype of lung tumors. We presented a case of SMARCA4-UT in a 50-year-old man with progressively worsening respiratory failure. The tumor was the first reported to involve pulmonary artery, and 90% of tumor cells expressed programmed cell death ligand 1 (PD-L1). High tumor mutational burden (TMB, 23.93/Mb) and mutations in SMARCA4 were detected. It is the first reported case to receive Tislelizumab monotherapy with considerable improvement in clinical condition and no adverse events. As a result of our case, we highlight the importance of recognizing SMARCA4-UT as an individual entity, as well as the efficacy of immune checkpoint inhibitor therapy, particularly in patients with high levels of TMB and PD-L1 expression.

## Introduction

Thoracic SMARCA4-deficient undifferentiated tumor (SMARCA4-UT), an aggressive and rare malignancy, is characterized by SMARCA4 gene inactivating mutation and presents with rapidly progressive masses involving the mediastinum, lung, and pleura. It has been established as a new histomorphological and molecular entity of thoracic tumors (1). Since the first report by Sauter et al. in 2015 up to now, no more than 100 cases have been reported in the literature (2).

There are no treatment guidelines for SMARCA4-UT. It was previously classified as a sarcoma subtype and treated with chemotherapy, which was often ineffective. Despite operable cases, recurrence occurs and systemic chemotherapy is required (2, 3). Recently, a few patients with SMARCA4-UT have been reported to be successfully treated with anti-PD-1 and PD-L1 antibodies (4–8).

We herein report a rapid response to Tislelizumab as the first-line treatment in a SMARCA4-UT with PD-L1 overexpression.

## Case presentation

**Figure 1:**
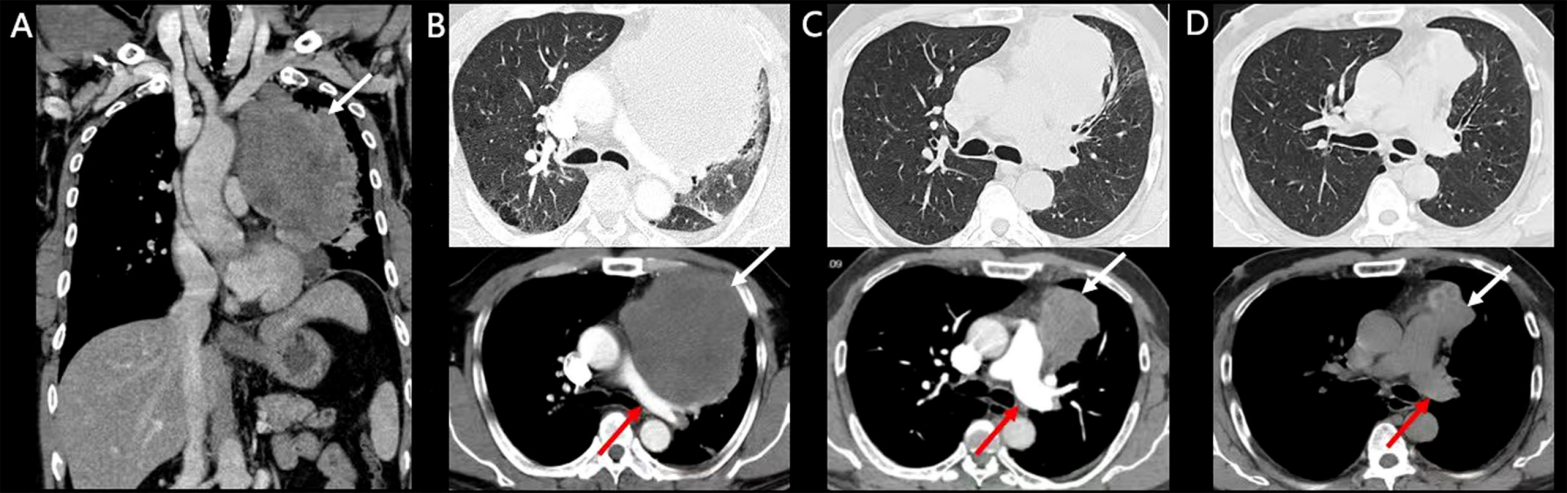
Enhanced CT and follow-up CT images. White arrows indicate tumors and red arrows indicate the pulmonary artery. **(A, B)** Pretreatment CT scan of the patient. **(C)** CT scan after two doses of Tislelizumab. **(D)** CT scan after six doses of Tislelizumab.

A 50-year-old man presented to our hospital complaining of increasingly worsening dyspnea, hemoptysis, and thoracic pain for 2 weeks with a weight loss of nearly 10 kg. He had a 36-pack-year smoking history and is a current smoker. There was no significant past medical history. His premorbid level of function was poor, with an Eastern Cooperative Oncology Group (ECOG) performance status (PS) of 3. His consciousness was clear; temperature was 36.2°C, blood pressure 136/88 mmHg, pulse 124/min, respiratory rate 26/min, and peripheral oxygen saturation 90% (room air). Following an examination of the respiratory system, decreased left-sided breath sounds were noted. Laboratory investigations revealed a normal white blood cell count of 6500/mm^3^ and a normal neutrophil count (71%; reference range, 40%–75%). Arterial blood gas showed a pH of 7.42, pCO_2_ of 32.8 mm Hg, and pO_2_ of 58 mm Hg. Assessment of serum tumor markers revealed a high neuron-specific enolase (NSE, 29.34 ng/ml; reference range ≤16.3), whereas carcinoembryonic antigen (CEA), cytokeratin 19 fragment (Cyfra 21-1), alpha-fetoprotein (AFP), and carbohydrate antigen 125 (CA125) were within the reference range. D-Dimer was elevated, 1.05 µg/ml (reference range, 0–0.5), and the evaluation of the cardiac function including B-type natriuretic peptide (BNP), Troponin I, and electrocardiogram was normal. A CT scan revealed a large mass of 11.46 cm × 11.19 cm in the left upper lobe of the lung (**Figure 1A**) involving the pulmonary artery and without enlargement of the hilar and mediastinal lymph nodes. Furthermore, thoracic and cardiac color Doppler ultrasound revealed a small amount of pleural and pericardial effusion. Cerebral, abdominal, and radionuclide bone scan examinations detected no abnormalities.

**Figure 2:**
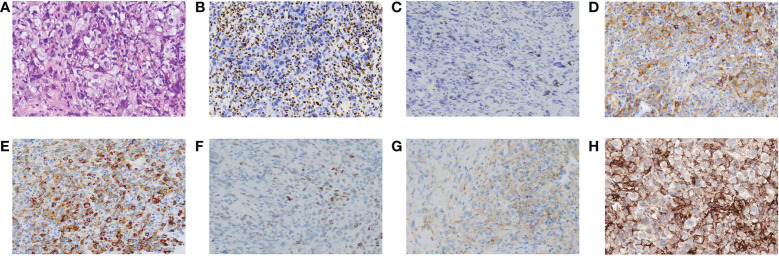
Pathological features of SMARCA4-UT. **(A)** Stained with hematoxylin and eosin. Immunostaining was negative for SMARCA4 **(B)**, and partly or focally positive for SMARCA2 **(C)**, CK8/18 **(D)**, CKpan **(E)**, SOX **(F)**, and Syn **(G)**. Programmed cell death ligand 1 (PD-L1) staining **(H)**. Magnification, 100×.

We performed a color Doppler ultrasound-guided percutaneous needle biopsy for the mass. The following clinicopathological examination revealed sheets of homogenous large tumor cells with a moderate amount of eosinophilic cytoplasm, eccentrically positioned vesicular nuclei, and prominent nucleoli (**Figure 2A**). On immunohistochemistry, the mass was completely SMARCA4-negative and partly positive for SMARCA2 (**Figures 2B, C**). These tumor cells were focally positive for cytokeratin (CK), CK7, CKpan, CK8/18, and Syn (**Figures 2D–G**) and diffusely positive for vimentin. They were negative for Claudin-4, Napsin-A, TTF-1, S-100, CK5/6, and CgA. In addition, Ki-67 staining was positive (60%). A 520-gene panel next-generation sequencing (NGS) was performed, which showed a somatic *SMARCA4* c.797C>T (p. Ser266*) mutation and no abnormal SMARCB1 gene. The tumor exhibited microsatellite stability (MSS), tumor mutational burden (TMB) of 23.93 muts/Mb, and high PD-L1 expression (tumor proportion score more than 90%) (**Figure 2H**) with concurrent mutations in *KEAP1*, *TP53*, and *TERT*. According to the diagnostic criteria of the WHO (1), all evidence resulted in a definitive diagnosis of stage IVa SMARCA4-UT (ECOG PS 3) involving the pulmonary artery, pericardium, and pleura.

**Figure 3:**
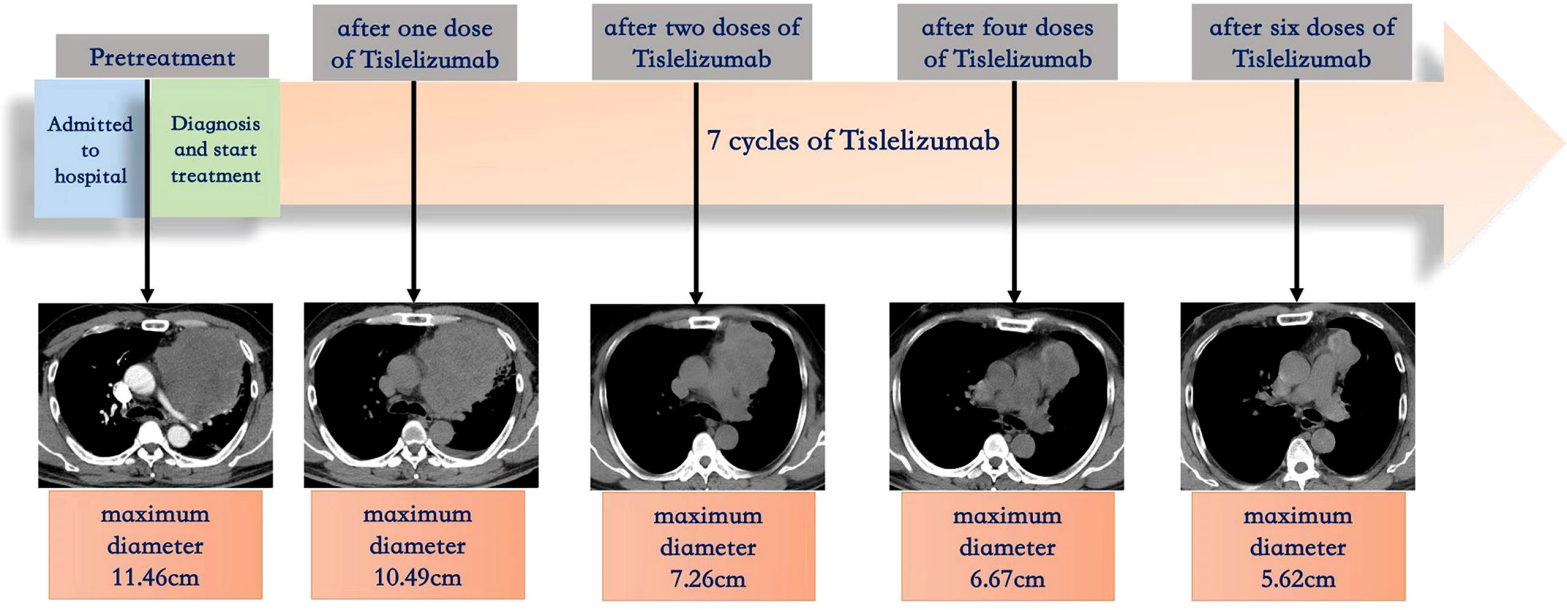
Treatment timeline and therapeutic response.

The patient was treated with Tislelizumab. After one dose of Tislelizumab (200 mg) infusion, the patient’s dyspnea, thoracic pain, and hemoptysis were rapidly relieved the next day, suggesting a notable clinical benefit. ECOG PS reduced from 3 to 1 with stable vital signs, and peripheral oxygen saturation rose to 96% (room air). Arterial blood gas showed a pH of 7.45, a pCO_2_ of 37.2 mm Hg, and a pO_2_ of 76 mm Hg. Regular CT scans revealed significant improvement in pulmonary artery compression and a partial response (PR; **Figures 1B, C**) after 20 days. A CT scan after six cycles of Tislelizumab demonstrated a sustained durable PR response [51% tumor size compared with baseline pretreatment according to RECIST version 1.1 (9)] with no adverse events (**Figure 1D**). The treatment timeline and therapeutic response are shown in **Figure 3**.

## Discussion

In recent years, SMARCA4-UT has become increasingly recognized as a progressive malignant disease. There has been an important change in terminology since the ‘sarcoma’ classification was dropped in favor of ‘thoracic SMARCA4-deficient undifferentiated tumor (SMARCA4-UT)’, which is newly classified in the fifth edition of the WHO classification of thoracic tumors (2021) (1). It was classified as a subtype of undifferentiated lung cancer of pulmonary epithelial origin (10). These tumors mostly occur in the fourth and fifth decades of life; moreover, there is a strong connection with smoking history (only 10% of the cases are reported in non-smokers). It often occurs in advanced stages, presenting a poor prognosis, with a median survival of 4–7 months (1, 4, 11). The clinical features of most cases present with non-specific symptoms such as cough, dyspnea, and hemoptysis (11). Some cases present with related signs like the superior vena cava syndrome (12) and distant metastasis; in particular, skeletal metastases are common (13). Recurrent pleural effusions or empyema can accompany pleural masses (14). Only one asymptomatic case has been reported, in a smoker during a routine chest X-ray (15).

Among thoracic SMARCA4-UTs, the involvement of the mediastinum, pulmonary hilum, lung, cervical-subclavian lymph nodes, and/or pleura is common with or without chest wall invasion. The common imaging findings of SMARCA4-UT presented as ill-defined, large, and compressive masses, especially in the mediastinum, but the primary lung tumor may rarely be small (1). Metastases are frequent at presentation with adrenal, bone, and brain involvement (16). In rare cases, there are unilateral, multifocal, or single masses involving the visceral and parietal pleura, occasionally extending to the soft tissue of the chest wall. An isolated chest wall tumor (14) and axillary lymphadenopathy (17) are rare conditions. In our present case, metastases were present in the pericardium and the pleura, and it is the first reported case to show the involvement of the pulmonary artery. As a result, we suggest that imaging findings of an infiltrative, compressive, heterogeneous, ill-defined, large thoracic mass should be suspicious for SMARCA4-UT.

Histologically, SMARCA4-UT consists of undifferentiated sheets of relatively homogenous, incohesive tumor cells with prominent nuclei and vesicular nucleoli. There may be uniformly epithelioid and rhabdoid cells with abundant eosinophilic cytoplasm and high mitotic rates in tumor cells. The complete loss of Brahma-related gene-1 (*BRG1*), which is encoded by the *SMARCA4* gene, is typical, and 25% of the cases show a severe reduction of expression (11, 17, 18). Most cases show loss of *SMARCA2* (*BRM*) staining (19). A variety of low-molecular-weight cytokeratins, epithelial membrane antigens, and neuroendocrine markers are also present in the tumors. CD34, SOX2, and/or SALL4 are also expressed in many cases. Across all cases, *SMARCA4* mutations are homozygous with loss of heterozygosity (10). The ATPase subunits of the *SWI/SNF* complex are encoded by the *SMARCA4* gene (17). A loss of *BRG1* leads to a failure of the ATPase-dependent eviction of polycomb recessive complexes, resulting in chromatin reorganization, as well as changes in the levels of gene expression that likely contribute to tumorigenesis. *BRM* deficiency may potentiate sarcomatoid transformation and epidermal mesenchymal transition (20). In general, the clinical presentation and radiological features of SMARCA4-UT are non-specific. It is essentially a clinicopathological diagnosis.

The current study on therapy for a new and challenging tumor entity is fragmented (21). Most cases present are generally resistant to chemotherapy and radiotherapy (19). In some surgically resectable cases, radical surgical excision has led to remission (5, 15). Novel treatment strategies are awaited, including immune checkpoint inhibitions (ICIs). ICIs had shown promising effects in some cases. Our literature review indicated that seven other cases have used immunotherapy for the treatment of SMRACA4-UT (4, 6–8, 15). In both low and high PD-L1 expression and effector T-cell signatures, immunotherapy has been shown to improve progression-free survival (PFS). The clinical data of patients under the treatment of immunotherapy are summarized in **Table 1**. The patients’ age ranged from 41 to 73 years, and the data exhibited a female-to-male ratio of nearly 1:1. All the patients had advanced-stage diseases. The longest PFS exhibited was more than 17 months, which was with ABCP treatment (7). In one case, renal dysfunction and respiratory failure progressively worsened in the patient, who died 25 months after the initial diagnosis (8). Six of seven cases combined immunotherapy with other treatments such as chemotherapy, radiotherapy, and even surgery (4, 7, 8, 15). A combination of pembrolizumab and ipilimumab produced mixed results in one study, which is the first case to use dual ICI combination therapy for SMARCA4-UT with PD-L1 overexpression (100%) (8). Only one case was given pembrolizumab monotherapy as a first-line treatment and demonstrated a sustained durable PR response after eight cycles (6).

**Table 1:** Clinical data for patients with SMARCA4-UT on the therapy of immunotherapy.

	No.	Age(Y)/gender	Smoking status	Lesion location	TMN	PD-L1 TPS (%)	TMB (/Mb)	Mutation	Treatment	Outcome (follow-up)
Henon et al. (4)	1	58/F	Unknown	Mediastinal, hilar and paratracheal involvement, along with pleural and peritoneal metastases	IV	0	Remarkably low	Unknown	Radiotherapy+ chemotherapy+ pembrolizumab	11 months PR to March 2019
Kunimass et al. (5)	2	51/M	22.5 pack-years	Right lung upper lobe with soft tissue, vertebral invasion and pleural	IVA	0	10.2	*SMARCA4*(L1161fs), *TP53* (V157L)	ABCP + surgery	9 months PR after surgery to August 2021
Kohichi et al. (6)	3	69/F	Unknown	Left mediastinal tumor, peritoneal and retroperitoneal dissemination and multiple cutaneous metastases	IV	60	Unknown	Unknown	Pembrolizumab	8 cycles of PR to September 2019
Kawachi et al. (7)	4	73/F	53 pack-years	Left upper lobe, mediastinal lymphadenopathy and osteolytic metastasis in the fifth cervical vertebra	IVB	40	11	*SMARCA4*(c.1119-1G>T), *TP53, SPTA1, CHD2*	ABCP	Progressed after 17 months of PR to 2021
Kawachi et al. (7)	5	59/M	39 pack-years	Left lower lobe, an oropharyngeal mass, left adrenal gland mass, left cervical lymphadenopathy, left hilar lymphadenopathy and multiple abdominal lymphadenopathies	IVB	0	11.8	*SMARCA4*(K755Nfs), *TP53*, *TSC2*	ABCP	Progressed after 10 months of PR to 2021
Kawachi et al. (7)	6	64/F	44 pack-years	Left upper lung lobe, mediastinal lymphadenopathy and a brain mass in the left parietal lobe	IVB	80	14.9	*SMARCA4*(H103Mfs), *TP53,SPTA1,TSC2*, *KEAP1*	ABCP+ Radiotherapy	Progressed after 7 cycles of PR and 2 months maintenance therapy to 2021
Anžiča et al. (8)	7	41/M	Current smoker	Upper anterior mediastinum encompassing infracarinal and hilar lymph nodes.	IV	100	674SNV and 47 indels	*CDKN2A*(p.T18fs), *SMARCA4*(p.K1334fs)	Pembrolizumab + ipilimumab + chemotherapy + radiotherapy	25 months to death

ABCP, atezolizumab, bevacizumab, paclitaxel, and carboplatins.

The mechanisms that determine the efficacy of immunotherapy are yet to be fully elucidated. A growing body of evidence suggests that PD-L1 and TMB expression play a role in this disease (10, 22). Three studies on respective patients showed improvement after treatment of PD-1 antibody (pembrolizumab) or PD-L1 antibody (atezolizumab) in patients with high PD-L1 expression (>50%) (6–8). Of note, three cases that did not express PD-L1 exhibited PFS longer than 10 months (4, 5, 7), and a sustained PR response with no adverse events was demonstrated. These cases suggest that there may be therapeutic effect regardless of the expression of PD-L1 and TMB. However, due to the limited number of cases, TMB and PD-L1 expression cannot yet be used as independent predictors of prognosis, and, therefore, further studies and exploration are required.

In one of these cases, although TMB and PD-L1 TPS were high, PFS was still the shortest with a combination therapy of ABCP and radiotherapy (8). Schoenfeld et al. (22) have evaluated the genomic context of *SMARCA4* alterations, which showed that *SMARCA4* alterations often co-occur with *TP53*(56%), *KEAP1*(41%), *STK11*(39%), and *KRAS* (36%) alterations in lung cancer. Co-occurrence of *STK11* and *KEAP1* mutations in SMARCA4-mutant NSCLC was associated with decreased survival. Further evaluation of factors that predict outcomes to ICIs revealed that *SMARCA4-*mutant tumors with *STK11* and *KEAP1* mutations have a poor prognosis and lack of response to ICIs in *KRAS*-mutant tumors. Further research by Marinelli et al. (23) has indicated the *KEAP1* mutation is associated with resistance to immunotherapy in lung adenocarcinoma despite the high TMB. This may suggest that the poor efficacy of immunotherapy in cases with high tumor mutational burden and PD-L1 expression may be related to *KEAP1* mutation. In contradiction, in our case, response was rapid despite *KEAP1* mutation detection. Overall, the role of *KEAP1* mutation in SMARCA4-UT remains unclear.

In our case, the tumor presented as a large compressive mass and is the first reported case to involve the pulmonary artery, pericardium, and pleura without extrathoracic metastasis. This case also showed overexpressed PD-L1 TPS of 90% and remarkably high TMB (29.93/Mb). It is the first reported case to receive Tislelizumab monotherapy with considerable and rapid improvement in clinical condition. Following seven courses of PD-1 inhibitor therapy, a CT scan demonstrated a sustained durable partial response with no adverse events.

In conclusion, the diagnosis of SMARCA4-UT is sometimes challenging. A multidisciplinary approach to diagnosis is essential, including clinical, radiologic, pathologic, and genetic factors. Our findings support the fact that ICIs may be beneficial, especially in patients with high PD-L1 expression and TMB. It is necessary to conduct further studies to optimize novel therapeutic approaches and to clarify whether ICI monotherapy or combination therapy is appropriate.

## Data availability statement

The raw data supporting the conclusions of this article will be made available by the authors, without undue reservation.

## Ethics statement

The studies involving human participants were reviewed and approved by Committee of the 2nd Affiliated Hospital of Fujian Medical University in China (ethical code B2021-315). The patients/participants provided their written informed consent to participate in this study. Written informed consent was obtained from the individual for the publication of any potentially identifiable images or data included in this article.

## Author contributions

LS and XC made accurate diagnosis and contributed to the care of this patient, the analysis of the clinical data and the literature review. LL and YD provided technical help or writing assistance. YZ was the departmental chair and provided general support. All authors contributed to the article and approved the submitted version.

## Funding

National Key Research and Development Program of China (2019YFC0121705); Quanzhou City Science and Technology Program (Award number(s): 2020N034S, 2019C025R, 2018N008S); Bethune Charitable Foundation (Award number(s):SCZ134DS); Startup Fund for Scientific Research, Fujian Medical University (Grant number: 2021QH1111).

## Conflict of interest

The authors declare that the research was conducted in the absence of any commercial or financial relationships that could be construed as a potential conflict of interest.

## Publisher’s note

All claims expressed in this article are solely those of the authors and do not necessarily represent those of their affiliated organizations, or those of the publisher, the editors and the reviewers. Any product that may be evaluated in this article, or claim that may be made by its manufacturer, is not guaranteed or endorsed by the publisher.
